# Mechanism of Electrochemical Delamination of Two-Dimensional Materials from Their Native Substrates by Bubbling

**DOI:** 10.3390/s151229888

**Published:** 2015-12-16

**Authors:** Jie Sun, Xing Fan, Weiling Guo, Lihui Liu, Xin Liu, Jun Deng, Chen Xu

**Affiliations:** 1 Key Laboratory of Optoelectronics Technology, College of Electronic Information and Control Engineering, Beijing University of Technology, Beijing 100124, China; fanguan123@126.com (X.F.); liuxin_f@163.com (X.L.), dengsu@bjut.edu.cn (J.D.); xuchen58@bjut.edu.cn (C.X.); 2 Quantum Device Physics Laboratory, Department of Microtechnology and Nanoscience, Chalmers University of Technology, Göteborg 41296, Sweden; lihui@chalmers.se

**Keywords:** graphene, chemical vapor deposition, electrochemical bubbling transfer

## Abstract

A capacitor-based circuit model is proposed to explain the electrochemical delamination of two-dimensional materials from their native substrates where produced gas bubbles squeeze into the interface. The delamination is actually the electric breakdown of the capacitor formed between the solution and substrate. To facilitate the procedure, the backside of the ubstrate has to be shielded so that the capacitor breakdown voltage can be reached. The screening effect can be induced either by nonreactive ions around the electrode or, more effectively, by an undetachable insulator. This mechanism serves as a guideline for the surface science and applications involving the bubbling delamination.

## 1. Introduction

Graphene, a monolayer of carbon atoms organized in a graphitic lattice, has emerged as a material with high potential in various aspects of futuristic electronics. The roadmap for graphene [1] predicts it to be commercialized as flexible transparent electrodes for optoelectronic devices within a few years, but graphene is just the tip of the iceberg when considering the large family of two-dimensional (2D) materials. For example, *h*-BN with its 6.15 eV bandgap is suitable for ultraviolet light emitting diodes. MoS_2_ is a semiconductor that can serve as the transistor channel material in post-silicon electronics. With modern technologies like chemical vapor deposition (CVD) [2], these atomic sheets are scalable and can be produced in large amounts: 100 m long graphene films has been reported [3]. Also, in-plane [4] and out-of-plane [5] 2D material heterostructures are realized. Increasingly sophisticated materials and devices can be designed with various combinations of 2D crystals and their stacking/connecting sequences, facilitating giant leaps in electronic device performance.

However, 2D materials are usually synthesized on expensive substrates such as ultrapure metal foils or silicon carbide, and need to be transferred to a target substrate for most applications and basic research, resulting in environmental and cost issues. For instance, graphene is grown by CVD on Cu, which needs to be etched off so that the graphene can be transferred to insulators. This poses a threat to the environment due to the heavy metal pollution, and also dramatically increases the raw materials cost. Therefore, it is necessary to develop a method to delaminate graphene directly from metals [6]. Recently, a transfer technique based on the mechanical separation of graphene from metal foils by H_2_ bubble formation at the cathode of a water electrolytic cell was proposed [7,8,9,10,11,12], where the catalyst foils are not consumed and can be recycled for re-growth of 2D materials. Apart from solving the aforementioned problems, this electrochemical delamination itself is also an excellent platform for surface science studies. To date, nevertheless, its seemingly simple mechanism is not well understood, although a detailed microscopic description of the hydrogen bubble nucleation and growth process is reported [12]. At a first glance, one might think the electrolyte is simply used to enhance the conductivity of H_2_O, but we find that not every electrolyte works in graphene delamination experiments, despite that fact that the solutions are all well conducting. The efficiency of the delamination is also heavily influenced by other factors like the flexibility of the mechanical support, sample size, *etc.*

This paper attempts to shed light on the complex mechanism of this electrochemical process. We studied experimentally the delamination process of a thin layer of spin-coated polymethyl methacrylate (PMMA) polymer from the surface of platinum foils. PMMA is commonly used as the mechanical support for 2D materials during transfer. However, in this specific work we firstly studied the case where there are no 2D materials between the PMMA and Pt, which can simplify and isolate the problem. Pt is chosen based on its chemical inertness, which also helps us to grasp the essence of the problem. The peeling off procedure is modeled as the breakdown of capacitors. We propose a screening mechanism for the open surfaces of Pt foils, which plays an important role in boosting the delamination efficiency. Anode procedures involving O_2_ bubbles, as well as chemical reaction other than water electrolysis are also studied. The as-developed mechanism can be easily applied to the actual bubbling transfer of almost all 2D materials. The theory can explain most experimental findings phenomenally. It will not only contribute to the understanding of this interesting surface science subject, but also serve as a guideline for real applications derived from the bubbling transfer of 2D materials.

## 2. Experimental Section 

**Figure 1:**
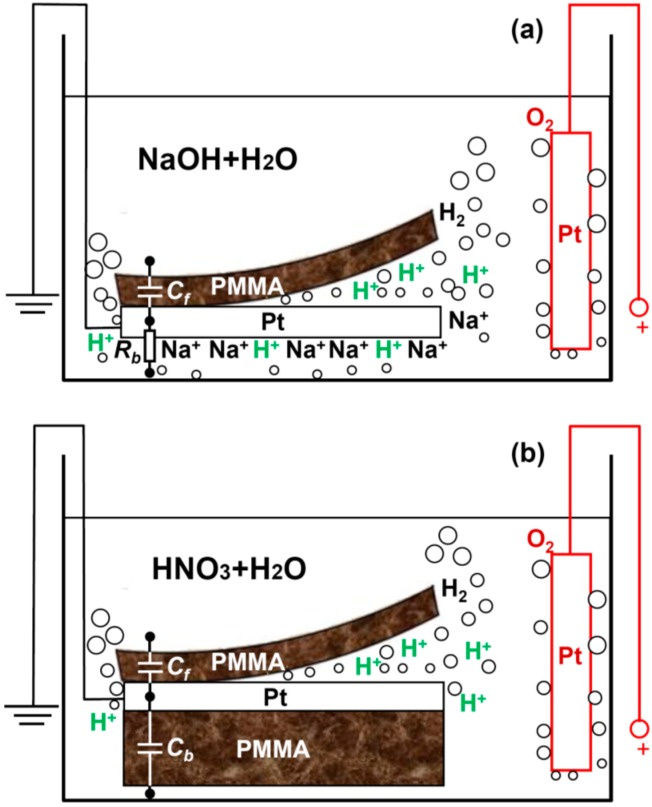
Schematic illustration of the bubbling delamination of a thin PMMA layer from its platinum substrate which is used as the cathode in a water electrolysis cell. To accelerate the process, screening effects against H^+^ reduction at the backside of the Pt are induced either by (**a**) non-reactive cation cloud of Na^+^ from the NaOH electrolyte or (**b**) an undetachable thick insulating PMMA block.

Figure 1a shows schematically a typical electrochemical delamination process. The Pt foil (2 cm × 2 cm, thickness 100 µm and purity 99.99%, purchased from the Beijing Nonferrous Metal and Rare Earth Research Institute, Beijing, China) is spin-coated with PMMA (950 A4, 1000 rpm, thickness~500 nm) and cured at 160 °C. Then, it is connected as the cathode in a water electrolysis cell. The anode is made of a bare Pt foil. The half equations of the reactions at the cathode and anode are

(1) 2H^+^ + 2e^−^ → H_2_, E^0^ = 0.00 V

(2) 2H_2_O → O_2_ + 4H^+^ + 4e^−^, E^0^ = 1.23 V

respectively, where E^0^ is the standard electrode potential and E^0^_cell_ = E^0^_cathode_ − E^0^_anode_ = −1.23 V. Since E^0^_cell_ < 0, this is an electrolytic cell (the process is non-spontaneous, as opposed to a galvanic cell), and the decomposition potential is 1.23 V. Starting from the sides, the H_2_ bubbles enter the interface between the PMMA and Pt. As soon as a small part is detached, the front of the peeling advances further until the PMMA is fully peeled off. Therefore, it is important to have a thin and flexible PMMA layer so as to be bendable (Figure 1a). Drop-coated mm-thick PMMA block is very stiff and cannot be effectively delaminated (see Figure 1b, backside of the Pt cathode).

## 3. Results and Discussion

**Figure 2:**
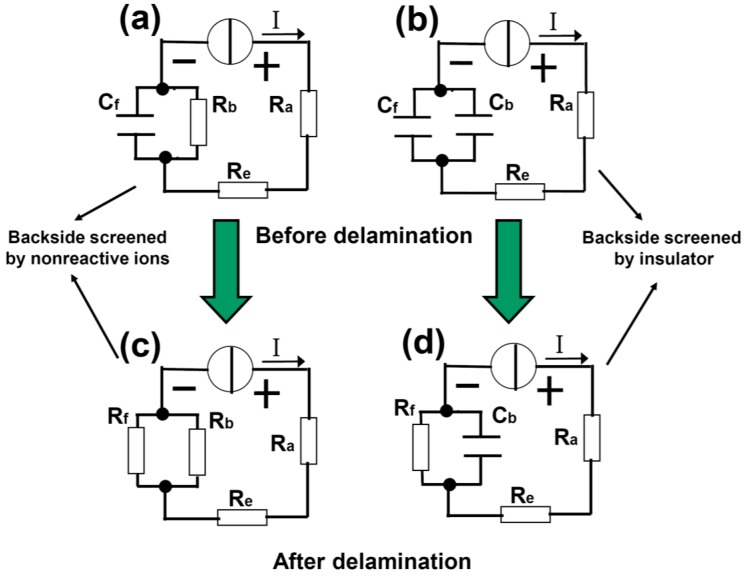
Capacitor model of the electrochemical delamination of 2D materials. (**a**,**b**) are before while (**c**,**d**) are after the delamination. The delamination is modeled by the C_f_ (formed between the solution and substrate) breakdown into R_f_, which is boosted by two types of screening effects from nonreactive ions and stable insulator at the backside of substrate.

In order to quantitatively understand the delamination process, we develop a capacitor model as shown in Figure 2. In Figure 2a,c, the front side of the Pt cathode is covered by spin-coated PMMA and the backside is open (sample type A). Figure 2a,c describes the case before and after the delamination, respectively. R_a_ and R_e_ are resistances associated with the anode and electrolytic solution. At the cathode, the *interface* resistance between the Pt backside and the solution is represented by R_b_. The front side can be regarded as a capacitor C_f_, formed between the Pt bulk and the electrolytic solution with the dielectric being the PMMA, see also Figure 1a. Note that the insulation resistance for capacitor C_f_ is not infinite because there is a path for leakage current at the Pt edge, but we will ignore this effect for simplicity. Also, the resistance of the bulk Pt is neglected. A constant current I is applied to the electrolysis cell (Figure 2a). As long as IR_b_ is greater than the critical breakdown voltage V_f0_ of the capacitor C_f_, the PMMA starts to peel off. In the end of the delamination, the front surface of the Pt is entirely open and the *interface* resistance to the solution is denoted by R_f_ (Figure 2c). Therefore, the bubbling separation process can be conveniently described by this lumped circuit model. Note that the breakdown of C_f_ does not occur across the dielectric, but rather along the PMMA. The PMMA-Pt interface is much easier to break down (*i.e.*, delamination) compared to the electric puncture of the PMMA itself. Figure 2b,d illustrates a similar situation as Figure 2a,c, but the open surface of the backside Pt is now covered with a thick and inflexible PMMA block (Figure 1b, sample type B). In this case, both sides are modeled by capacitors (C_f_ and C_b_). With the voltage increasing, the bubbles attack C_f_ first, leading to the (interface) breakdown of the capacitor. After delamination, R_f_ is used to describe the Pt-solution interface resistance, whereas C_b_ which is very difficult to break down remains basically unchanged.

**Figure 3:**
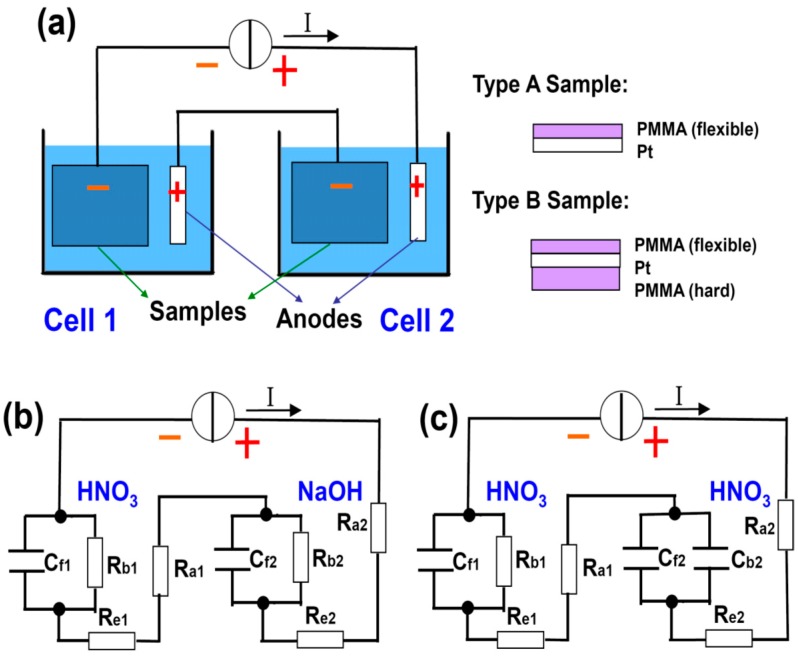
(**a**) Schematic drawing of the setup for comparison study of two electrochemical cells connected in series so that the amounts of generated bubbles at cathodes are equal. Two types of samples are used in the experiments; (**b**,**c**) are the equivalent circuits for test 1 and 2, respectively (see Table 1).

Clearly, the efficiency for delamination is determined by how fast the breakdown of C_f_ takes place. Now we will experimentally demonstrate that a screening effect on the backside Pt open surface can greatly boost the delamination of the front side thin PMMA by accelerating the speed to reach the critical breakdown voltage of C_f_. This screening effect can be induced by: (1) large numbers of non-reactive ions, e.g., Na^+^, surrounding the Pt backside as a “cloud” (Figure 1a), or (2) an insulator such as drop-coated thick PMMA block physically covering the Pt backside (Figure 1b). Figure 3a shows the schematic diagram of our setup, where the PMMA delamination time in two water electrolysis cells are compared. They are connected in series to a constant current source to ensure that they have equal currents and hence equal amount of H_2_ bubbles generated (assuming 100% current efficiency). This configuration facilitates our experimental observation, allowing the direct comparison of the delamination on site. Table 1 lists the parameters used, where tests 1 and 2 are designed to examine the screening effects from nonreactive cations and insulator, respectively. HNO_3_ and NaOH are used as the electrolytes. A monobasic acid (e.g., HNO_3_) is chosen instead of polybasic acid (e.g., H_2_SO_4_) because at the same concentration it releases an equal amount of cations as NaOH, which is a monobasic base. The concentration and volume of the solutions are fixed at 0.25 mol/L and 200 mL. A constant current of 0.5 A is applied to the systems. Two types of samples (A and B), as schematically drawn in Figure 3a, are used as the cathode in the two cells (see Table 1).

Based on Figure 2, it can be seen that during the breakdown process of C_f_, the total voltage drop across the electrolysis cell (between “+” and “−”) decreases and reaches its minimum when the delamination ends [13]. Together with direct eye observation, they are used as the criteria to determine the completion of the delamination. The equivalent circuits for tests 1 and 2 are shown in Figure 3b,c. In test 1, cell 1 contains many H^+^ ions primarily generated by the ionization of HNO_3_, which can be easily reduced at the uncovered backside of Pt foil. In cell 2, however, the majority cations that contribute to the conductivity are Na^+^ from NaOH. Even though they do not participate in the redox reaction, they carry the current and migrate towards the cathode. As a result, the gathering of Na^+^ shields the backside of the Pt cathode to some extent, hindering H^+^ from being reduced to H atoms by generating a large Coulomb repulsive force. Therefore, many H^+^ are forced to squeeze into the interface between PMMA and Pt and accept electrons therein. As the reaction front advances towards the interior, the bubbles eventually separate the two materials. The cloud of Na^+^ hence offers a screening effect against H^+^ at the Pt back, serving as the driving force for the hydrogen to form a wedge between the partly exfoliated PMMA and free Pt surface, and the subsequent continuous widening of this wedge. When the Na^+^ accumulation reaches its steady stage, the freshly arrived Na^+^ are neutralized by the OH^−^ produced by water decomposition 2H_2_O + 2e^−^ = H_2_ + 2OH^−^ and the net Na^+^ density is thus stabilized and stops increasing. In Figure 3b, the difference between the two cells in terms of the screening effect can also be understood as R_b1_ << R_b2_. At the given current I, the voltage IR_b2_ on C_f2_ is high enough to trigger the breakdown of C_f2_, whereas IR_b1_ is too small and hardly reaches the threshold breakdown voltage of C_f1_. Indeed, in experiments we observe that it takes approximately 2 min for the PMMA delamination to complete in cell 2, which never happens in cell 1 within 5 min. The role for an appropriate electrolyte is not simply boosting the conductivity of solution, but providing the driving force to bubble off of the PMMA as well.

**Table 1:** Summary of the electrochemical delamination comparison experiments using H_2_ bubbles.

		Electrolysis Cell 1	Electrolysis Cell 2	Constant Current (A)
Test 1	Electrolyte	HNO_3_	NaOH	0.5
	(aq. solution, 0.25 mol/L, 200 mL)			
	Sample at cathode	Type A	Type A	
Test 2	Electrolyte	HNO_3_	HNO_3_	0.5
	(aq. solution, 0.25 mol/L, 200 mL)			
	Sample at cathode	Type A	Type B	

**Figure 4:**
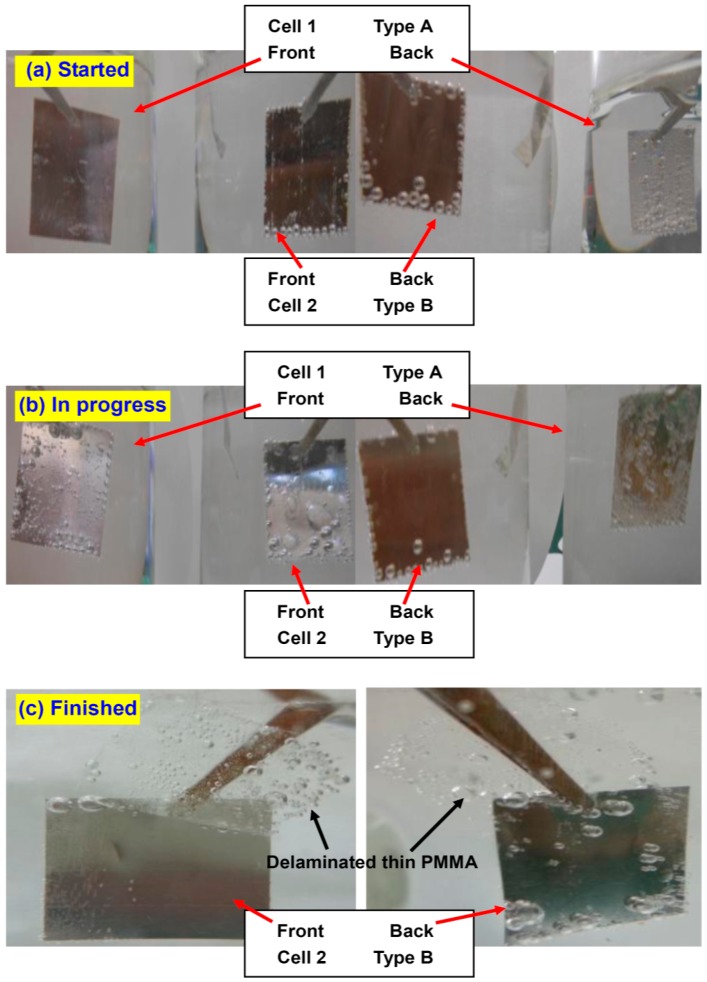
Photos of the samples where the experiment is (**a**) just started; (**b**) in progress and (**c**) already finished. The front side thin PMMA layer is entirely peeled off from the type B sample in cell 2 whereas remains undetached on the type A sample in cell 1. It is explained by the screening effect induced by the PMMA block at the backside of type B sample.

With this understanding, we can intentionally design an even more effective screening. In test 2, both solutions are HNO_3_, but the sample in cell 2 is changed to type B (Table 1 and Figure 3c). The backside of the type B sample is covered with a drop-coated PMMA block. It is inflexible and renders it almost impossible for a wedge to form at its interface to Pt. Therefore, it is supposed to entirely screen out H^+^ at the back and force them to be reduced at the interface between the thin/flexible front side PMMA and the Pt. This means R_b2_ will be dramatically increased (to infinity ideally) in Figure 3c, which is denoted by C_b2_ instead. Other parameters remain unchanged with respect to test 1. Figure 4 summarizes the photos of the samples taken at different stages. At the early stage of the procedure (5 s after power-on), the bubbles on type A sample in cell 1 dominantly appear on the backside, with the front side free of bubbles (Figure 4a). On the contrary, for type B sample, the bubbles gather mainly on the front, whereas there are almost no bubbles on the back, except at sample edges. After 2 min of electrolysis, a few bubbles can be seen on the front side of type A sample, but the majority are still on the backside (Figure 4b). On the other hand, the bubbles on the front side of type B sample already have evolved into large bubbles and the delamination is almost finished; the backside is still clear. Finally, after 5 min, type A sample is almost not exfoliated at all, due to the lack of screening from either passive cations or a stable insulator. In contrast, the PMMA film at the front side of type B sample has long since detached entirely (Figure 4c). The backside PMMA is very thick and inflexible, generating a good screening effect. This PMMA keeps attaching to the surface. 

The screening theory seems to dovetail well with the experimental findings. Microscopically, however, is the amount of Na^+^ really enough to create such a screening effect? When there is no current, on the Pt surface in 0.25 mol/L NaOH, the average distance between Na^+^ ions $d_{Na^{+}}$ is estimated to be 1.9 nm. During the electrolysis, if all the Na^+^ ions in the 0.2 L solution is attracted and condensed on the backside Pt surface (2 cm × 2 cm), $d_{Na^{+}}\;$ is then 0.15 pm. Of course, this calculated extreme case will never happen, but it gives an indication that the total amount of Na^+^ under our condition is several orders of magnitude more than enough to provide a screening effect against H^+^. In reality, some limited amount of H_2_ bubbles can still generate on the backside, unless it is perfectly shielded by an insulator that is stable enough to survive the bubbling delamination process.

Oxygen bubbles can also be used to separate the films, which is also heavily affected by the screening effect. We have done a series of experiments listed in Table 2. Compared with Table 1, here all samples are used as the anodes, and in test 4 the electrolytes are changed to NaOH. Other parameters are kept unchanged. Not surprisingly, in test 3, the delamination in HNO_3_ is far more efficient than in NaOH. It can be readily explained by the screening effect from the nonreactive NO_3_^−^ against OH^−^ anions which are sources for O_2_ generation. In test 4, type B sample works much better than type A sample due to the screening effect from the thick and nonflexible PMMA insulator at the back. Even though the screening theory works perfectly in predicting the experimental results for tests 3 and 4, however, we also notice that tests 1, 2 and tests 3, 4 are not entirely symmetric. In general, to delaminate a thin film, it takes 3–4 times longer time using O_2_ bubbles than using H_2_ bubbles. We believe the reasons for the deficiency of O_2_ bubbles in the exfoliation are three folds. First, the O_2_ evolution reaction at the anode is much more complicated and less efficient than the H_2_ evolution at the cathode. The exchange current densities for water oxidation on the best known catalysts RuO_2_ is 2–3 orders of magnitude lower than that for H_2_ production, meaning that these anodes generally operate at high overpotentials [14]. Also, most transition metals are catalysts for H_2_ evolution (can be simplified as H + H = H_2_; Pt is the best known catalyst in particular), but not O_2_ evolution [14]. Second, the radii of OH^−^ is 1.4 Å [15,16], several times larger than 0.4 Å of H^+^ (here we use Shannon’s “effective ionic radius” values where the hydrated ion enlargement effect is taken into account) [15]. The considerably greater volume makes it more difficult for OH^−^ to intercalate into the interface. Also, it is known that protons have an unconventionally high mobility in solutions due to the proton hopping mechanism as well as their low mass, which further enhances the H^+^ intercalation. Third, based on Equations (1) and (2), for the same amount of charge transfer, the mole ratio between O_2_ and H_2_ is 1/2. Of course, this will translate to the strength for the delamination accordingly.

**Table 2:** Summary of the electrochemical delamination comparison experiments using O_2_ bubbles.

		Electrolysis Cell 1	Electrolysis Cell 2	Constant Current (A)
Test 3	Electrolyte	HNO_3_	NaOH	0.5
	(aq. solution, 0.25 mol/L, 200 mL)			
	Sample at anode	Type A	Type A	
Test 4	Electrolyte	NaOH	NaOH	0.5
	(aq. solution, 0.25 mol/L, 200 mL)			
	Sample at anode	Type A	Type B	

**Figure 5:**
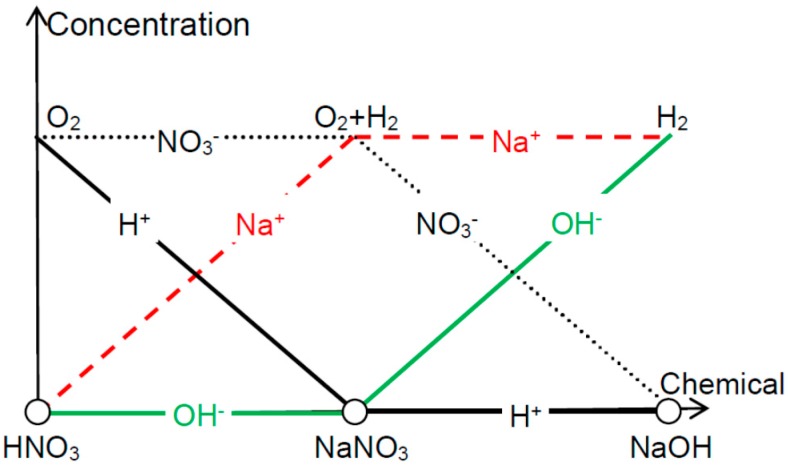
Depending on the acidity or alkalinity of the water electrolysis solution, oxygen or hydrogen bubbles are effective in delaminating the thin films from their substrates (with uncovered backside).

For type A samples, Figure 5 summarizes the effectiveness of exfoliation when using different electrolytes. In HNO_3_ solution, NO_3_^−^ ions can screen OH^−^ at the backside of the metal foils and hence O_2_ is peeling off the front side thin films. In NaOH, which is the other extreme, Na^+^ screens H^+^ so that H_2_ can be used for the detaching. In the case of NaNO_3_, both OH^−^ and H^+^ are shielded and therefore O_2_ and H_2_ both contribute to the delamination (though H_2_ is more effective due to the reasons listed above).

Other transitional situations, e.g., mixture, of HNO_3_ and NaNO_3_, can also be analyzed via relative weight analysis. Note that the relation [H^+^]∙[OH^−^] = K_w_ where K_W_ is the self-ionization constant of water cannot be reflected in this figure when [H^+^] and [OH^−^] are pushed towards the abscissa axis. Now let us look into the delamination efficiency when using reactions other than H_2_O electrolysis. The Kolbe electrolysis is decarboxylative dimerisation of two carboxylic acids (or carboxylate ions):

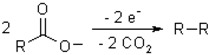
such as 2CH_3_COONa + 2H_2_O = C_2_H_6_ + 2CO_2_ + 2NaOH + H_2_ (the effective ionic radii for COOH^−^ is 1.6 Å) [16]. At a first glance, because the electrolyte CH_3_COONa is also taking part in the redox reaction, one may expect it to give a better delamination efficiency. Also, at the anode, there are two types of gases (C_2_H_6_ and CO_2_) with the total mole ratio to cathode H_2_ being 3:1. Nevertheless, in experiments we find that the delamination efficiency at the anode is very poor (200 mL CH_3_COONa solution at 0.25 mol/L), much worse than that for HNO_3_ under the same conditions. This is because the solution is dominated by CH_3_COO^−^ which is reactive (unlike NO_3_^−^) and cannot offer a screening effect.

For simplicity, the discussion above only involves the delamination of bare PMMA thin films. Actually, we have repeated the experiments on Pt-grown graphene samples (spin-coated with a thin PMMA layer) and exactly the same screening effects are observed [17]. Similar findings are found on Cu-grown graphene (although only connected as the cathode because Cu-anode will be electrochemically etched) [17]. As we know, other interesting stable monolayers such as *h*-BN, MoS_2_, *etc.* also demand a film transfer technology. We believe the screening effect is quite general and should be considered in the electrochemical delamination of all 2D materials beyond graphene. The bubbling separation process and the associated screening effect not only provide an ideal platform for surface science studies involving 2D materials, but also lead to economic and environmental benefits when it comes to applications. The metal foil catalysts used for 2D material growth are entirely reusable, drastically reducing the cost on raw materials and the heavy metal pollution. In the Supplementary Material, we propose a roll-to-roll industrial process for the bubbling delamination of 2D materials from their metal catalysts, with the screening effect discussed above taken into account. Also, our speculation on the bubbling delamination of graphene from insulating SiC substrate is presented. This type of graphene is often called epitaxial graphene [18,19,20,21], where a reliable transfer of the graphene is highly desired.

## 4. Conclusions

In summary, we have developed a capacitor-based lumped circuit model to explain the mechanism of electrochemical delamination of 2D materials from their native substrates. The hydrogen or oxygen gas bubbles produced by water electrolysis are directed to the interface between the 2D materials and substrates and separate them. The delamination is in fact the electric breakdown of the capacitor formed between the solution and substrate with the thin polymeric support being the dielectric. We have discovered that if the substrate backside is screened against the active ions that are contributing to the detachment, the thin films are much easier and faster to exfoliate. In other words, the capacitor’s critical breakdown voltage V_f0_ can be reached more easily. This shielding effect can be created by nonreactive ionic cloud adjacent to the electrode and/or, preferably, by an undetachable insulator on the back of the substrate. The proposed mechanism will be essential for the emerging 2D material surface science and industrial applications derived from the bubbling delamination technological route.

## Supplementary Files

Supplementary File 1

## References

[1] Novoselov K.S., Fal’ko V.I., Colombo L., Gellert P.R., Schwab M.G., Kim K. (2012). A Roadmap for graphene. Nature.

[2] Sun J., Lindvall N., Cole M.T., Angel K.T.T., Wang T., Teo K.B.K., Chua D.H.C., Liu J., Yurgens A. (2012). Low partial pressure chemical vapor deposition of graphene on copper. IEEE Trans. Nanotechnol..

[3] Kobayashi M., Bando M., Kimura N., Shimizu K., Kadono K., Umezu N., Miyahara K., Hayazaki S., Nagai S., Mizuguchi Y. (2013). Production of a 100-m-long high-quality graphene transparent conductive film by roll-to-roll chemical vapor deposition and transfer process. Appl. Phys. Lett..

[4] Levendorf M.P., Kim C.J., Brown L., Huang P.Y., Havener R.W., Muller D.A., Park J. (2012). Graphene and boron nitride lateral heterostructures for atomically thin circuitry. Nature.

[5] Geim A.K., Grigorieva I.V. (2013). Van der Waals heterostructures. Nature.

[6] Yoon T., Shin W.C., Kim T.Y., Mun J.H., Kim T.-S., Cho B.J. (2012). Direct measurement of adhesion energy of monolayer graphene as-grown on copper and its application to renewable transfer process. Nano Lett..

[7] Wang Y., Zheng Y., Xu X., Dubuisson E., Bao Q., Lu J., Loh K.P. (2011). Electrochemical delamination of CVD-grown graphene film: Toward the recyclable use of copper catalyst. ACS Nano.

[8] Gao L.B., Ren W.C., Xu H.L., Jin L., Wang Z.X., Ma T., Ma L.P., Zhang Z.Y., Fu Q., Peng L.M. (2012). Repeated growth and bubbling transfer of graphene with millimetre-size single-crystal grains using platinum. Nat. Commun..

[9] de la Rosa C.J.L., Sun J., Lindvall N., Cole M.T., Nam Y., Löffler M., Olsson E., Teo K.B.K., Yurgens A. (2013). Frame assisted H_2_O electrolysis induced H_2_ bubbling transfer of large area graphene grown by chemical vapor deposition on Cu. Appl. Phys. Lett..

[10] Zhan Z., Sun J., Liu L., Wang E., Cao Y., Lindvall N., Skoblin G., Yurgens A. (2015). Pore-free bubbling delamination of chemical vapor deposited graphene from copper foils. J. Mat. Chem. C.

[11] Ciuk T., Pasternak I., Krajewska A., Sobieski J., Caban P., Szmidt J., Strupinski W. (2013). Properties of chemical vapor deposition graphene transferred by high-speed electrochemical delamination. J. Phys. Chem. C.

[12] Fisichella G., di Franco S., Roccaforte F., Ravesi S., Giannazzo F. (2014). Microscopic mechanisms of graphene electrolytic delamination from metal substrates. Appl. Phys. Lett..

[B13-sensors-15-29888] 13.If a constant voltage source is used instead of a constant current source, then the current in the circuit increases and stabilizes at its maximum value when the delamination finishes. Unpublished Results.

[14] Walter M.G., Warren E.L., McKone J.R., Boettcher S.W., Mi Q.X., Santori E.A., Lewis N.S. (2010). Solar water splitting cells. Chem. Rev..

[15] Shannon R.D. (1976). Revised effective ionic-radii and systematic studies of interatomic distances in halides and chalcogenides. Acta Cryst. A.

[16] Manku G.S. (1980). Theoretical Principles of Inorganic Chemistry.

[17] Liu L., Liu X., Zhan Z., Guo W., Xu C., Deng J., Chakarov D., Hyldgaard P., Schröder E., Yurgens A. A mechanism for highly efficient electrochemical bubbling delamination of CVD-grown graphene from metal substrates. Adv. Mat. Interf..

[18] Maassen T., van den Berg J.J., IJbema N., Fromm F., Seyller T., Yakimova R., van Wees B.J. (2012). Long spin relaxation times in wafer scale epitaxial graphene on SiC(0001). Nano Lett..

[19] Gorantla S., Bachmatiuk A., Hwang J., Alsalman H.A., Kwak J.Y., Seyller T., Eckert J., Spencere M.G., Rummeli M.H. (2014). A universal transfer route for graphene. Nanoscale.

[20] Vecchio C., Sonde S., Bongiorno C., Rambach M., Yakimova R., Raineri V., Giannazzo F. (2011). Nanoscale structural characterization of epitaxial graphene grown on off-axis 4H-SiC (0001). Nanosc. Res. Lett..

[21] Nicotra G., Deretzis I., Scuderi M., Spinella C., Longo P., Yakimova R., Giannazzo F., La Magna A. (2015). Interface disorder probed at the atomic scale for graphene grown on the C face of SiC. Phys. Rev. B.

