# Supplementary material for: Mechanism of Electrochemical Delamination of Two-Dimensional Materials from Their Native Substrates by Bubbling
Source: Sensors (Basel). 2015 Dec 16;15(12):31811–20. doi: 10.3390/s151229888 (PMC4721807; doi:10.3390/s151229888)
Supplement: Supplementary File 1 [file sensors-15-29888-s001.pdf]

Mechanism of Electrochemical Delamination of Two-Dimensional Materials from Their Native Substrates by Bubbling

Jie Sun, Xing Fan, Weiling Guo, Lihui Liu, Xin Liu, Jun Deng and Chen Xu

Figure S1 shows a proposed roll-to-roll industrial process in the electrochemical delamination of 2D materials from their native metal foil substrates. Both sides of the metal foil are coated with a thin and flexible organic supporting film (such as a polyethylene terephthalate tape). The foil is immersed in a NaOH aqueous solution as the cathode. Importantly, the area between the two rollers in the electrolysis solution should be kept reasonably small. It is in this area that the bubbling delamination occurs. If it is too large, the freshly exposed open surface of the metal will reduce the delamination efficiency. Also, too large a cathode inevitably results in a nonnegligible resistance, which in turn leads to a considerable voltage drop along the foil and nonuniform detachment is to take place. H_2 and O_2 byproducts are collected at the electrodes. In Figure S1, reduced open surfaces, optimized electrolyte providing ionic screening effect and scalable roll-to-roll process help to take full advantage of the electrochemical delamination process, which is expected to be the standard technology route for substrate recycling and device processing of 2D materials.

Inspired by the experiments on type B samples, it is reasonable to argue that graphene on insulating substrates would provide the best delamination efficiency because reaction is allowed to take place only at the interface. Therefore, epitaxial graphene on insulating SiC is an ideal system. Such graphene is produced by heating SiC wafers to >1100 °C to evaporate the Si element on the surface. It is known that graphene grown on Si-face SiC is situated on a graphene buffer layer [1]. It is called a buffer because this graphene is insulating due to its covalent bonds to SiC. Since the "real" graphene is usually *n*-doped (~10^{12} cm^{-2}) and well conducting, we anticipate that the bubbles generate at its interface with the buffer. On C-face SiC, there is no buffer and the graphene is usually multilayers, which are expected to be transferred altogether. A hydrogenation treatment [2] which leads to the hydrogen intercalation and breaking of Si–C covalent bonds between the graphene and the SiC may be favorable to the exfoliation. However, SiC can hardly catalyze the H_2 evolution reaction, which might limit the delamination efficiency. The graphene also needs to be properly contacted so ensure a successful bubbling process. Finally, we note that on top of the discussion here, the microscopic mechanism of the bubbling transfer e.g. wrinkles in the film as the nucleation sites for H_2 bubbles [3] should be also considered when designing the actual experiment. Indeed, wrinkles (typically induced during the cooling down procedure after the growth) are present in epitaxial graphene on SiC, which is a result of the mismatch of thermal expansion coefficients between the graphene and substrate [4,5].

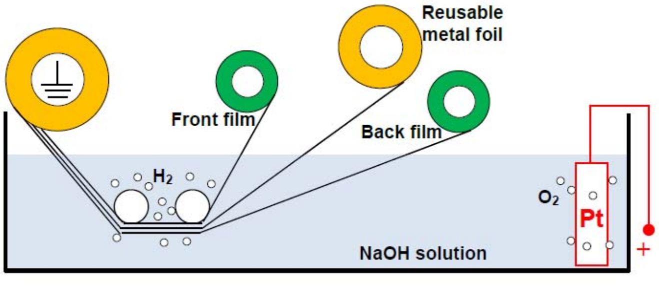

Figure S1. Proposed roll-to-roll scalable technology of the electrochemical delamination of 2D materials, where films on two sides of the catalyst foil are simultaneously peeled off by optimized bubbling process at the cathode.

References (*i.e.*, Refs. 12 and 18–21 in the main text)

1. Maassen, T.; van den Berg, J.J.; IJbema, N.; Fromm, F.; Seyller, T.; Yakimova, R.; van Wees, B.J. Long spin relaxation times in wafer scale epitaxial graphene on SiC(0001). *Nano Lett.* **2012**, *12*, 1498–1502.
2. Gorantla, S.; Bachmatiuk, A.; Hwang, J.; Alsalman, H.A.; Kwak, J.Y.; Seyller, T.; Eckert, J.; Spencere, M.G.; Rummeli, M.H. A universal transfer route for graphene. *Nanoscale* **2014**, *6*, 889–896.
3. Fisichella, G.; Di Franco, S.; Roccaforte, F.; Ravesi, S.; Giannazzo, F. Microscopic mechanisms of graphene electrolytic delamination from metal substrates. *Appl. Phys. Lett.* **2014,** *104*, doi:10.1063/1.4882165.
4. Vecchio, C.; Sonde, S.; Bongiorno, C.; Rambach, M.; Yakimova, R.; Raineri, V.; Giannazzo, F. Nanoscale structural characterization of epitaxial graphene grown on off-axis 4H-SiC (0001). *Nanosc. Res. Lett.* **2011,** *6*, doi.:10.1186/1556-276X-6-269.
5. Nicotra, G.; Deretzis, I.; Scuderi, M.; Spinella, C.; Longo, P.; Yakimova, R.; Giannazzo, F.; La Magna, A. Interface disorder probed at the atomic scale for graphene grown on the C face of SiC. *Phys. Rev. B* **2015,** *91*, doi:10.1103/PhysRevB.91.155411.
